# Polymorphisms of *TNF*-enhancer and gene for FcγRIIa correlate with the severity of falciparum malaria in the ethnically diverse Indian population

**DOI:** 10.1186/1475-2875-7-13

**Published:** 2008-01-14

**Authors:** Swapnil Sinha, Shrawan K Mishra, Shweta Sharma, Phani K Patibandla, Prashant K Mallick, Surya K Sharma, Sanjib Mohanty, Sudhanshu S Pati, Saroj K Mishra, Bheshaj K Ramteke, RM Bhatt, Hema Joshi, Aditya P Dash, Ramesh C Ahuja, Shally Awasthi, Indian Genome Variation Consortium, Vimala Venkatesh, Saman Habib

**Affiliations:** 1 Division of Molecular and Structural Biology, Central Drug Research Institute, Post box 173, Chattar Manzil, Mahatma Gandhi Marg, Lucknow-226001, India; 2 Department of Microbiology, King George Medical University (KGMU), Lucknow, India; 3 National Institute of Malaria Research (NIMR), New Delhi, India; 4 NIMR Field Station, Rourkela, India; 5 Department of Internal Medicine, Ispat General Hospital, Rourkela, India; 6 Department of Biochemistry, Ispat General Hospital, Rourkela, India; 7 Community Health Centre, Antagarh, India; 8 NIMR Field Station, Raipur, India; 9 Department of Medicine, KGMU, Lucknow, India; 10 Department of Paediatrics, KGMU, Lucknow, India; 11 Institute of Genomics and Integrative Biology, Delhi, India

## Abstract

**Background:**

Susceptibility/resistance to *Plasmodium falciparum *malaria has been correlated with polymorphisms in more than 30 human genes with most association analyses having been carried out on patients from Africa and south-east Asia. The aim of this study was to examine the possible contribution of genetic variants in the *TNF *and *FCGR2A *genes in determining severity/resistance to *P. falciparum *malaria in Indian subjects.

**Methods:**

Allelic frequency distribution in populations across India was first determined by typing genetic variants of the *TNF *enhancer and the *FCGR2A *G/A SNP in 1871 individuals from 55 populations. Genotyping was carried out by DNA sequencing, single base extension (SNaPshot), and DNA mass array (Sequenom). Plasma TNF was determined by ELISA. Comparison of datasets was carried out by Kruskal-Wallis and Mann-Whitney tests. Haplotypes and LD plots were generated by PHASE and Haploview, respectively. Odds ratio (OR) for risk assessment was calculated using EpiInfo™ version 3.4.

**Results:**

A novel single nucleotide polymorphism (SNP) at position -76 was identified in the *TNF *enhancer along with other reported variants. Five *TNF *enhancer SNPs and the *FCGR2A *R131H (G/A) SNP were analyzed for association with severity of *P. falciparum *malaria in a malaria-endemic and a non-endemic region of India in a case-control study with ethnically-matched controls enrolled from both regions. *TNF *-1031C and -863A alleles as well as homozygotes for the TNF enhancer haplotype CACGG (-1031T>C, -863C>A, -857C>T, -308G>A, -238G>A) correlated with enhanced plasma TNF levels in both patients and controls. Significantly higher TNF levels were observed in patients with severe malaria. Minor alleles of -1031 and -863 SNPs were associated with increased susceptibility to severe malaria. The high-affinity IgG2 binding FcγRIIa AA (131H) genotype was significantly associated with protection from disease manifestation, with stronger association observed in the malaria non-endemic region. These results represent the first genetic analysis of the two immune regulatory molecules in the context of *P. falciparum *severity/resistance in the Indian population.

**Conclusion:**

Association of specific *TNF *and *FCGR2A *SNPs with cytokine levels and disease severity/resistance was indicated in patients from areas with differential disease endemicity. The data emphasizes the need for addressing the contribution of human genetic factors in malaria in the context of disease epidemiology and population genetic substructure within India.

## Background

The association of severity of malaria with several human genetic factors is well documented [1] and the disease has been the selective pressure behind several erythrocytic defects such as sickle cell disease, G6PD deficiency and thalassaemia [2,3]. Malaria susceptibility/resistance has been correlated with polymorphisms in more than 30 other genes, some of which have exhibited differential association in distinct populations of the world [1]. *Plasmodium falciparum *blood infection levels and fever episodes have been linked to chr 5q31–33 [4] and chr10 [5]. While most human gene polymorphism-disease association studies for malaria susceptibility have been carried out on populations from Africa and south-east Asia, there is limited information on malaria-associated gene polymorphisms in Indian populations [6-8].

**Figure 1:**
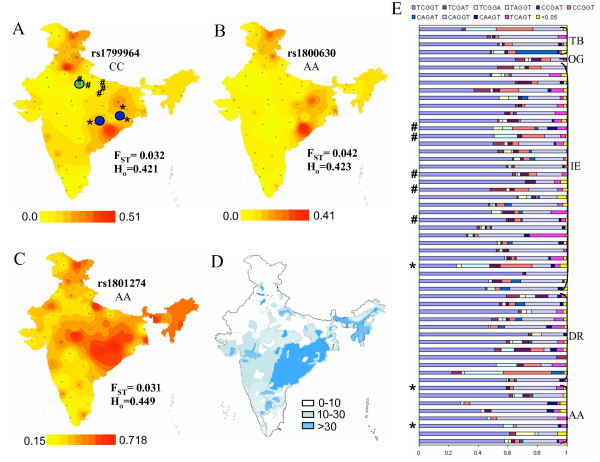
Gradient maps representing genotype frequency distribution of the minor allele of *TNF *promoter/enhancer and *FCGR2A *G/A R131H SNPs across India. A, rs1799964 (-1031T>C); B, rs1800630 (-863C>A); C, rs1801274 (exon4 G>A). Dots on each map depict the 55 IGVC endogamous populations. H_o _and F_ST _value of each SNP is indicated. D, map depicting *P. falciparum *malaria prevalence across the country as percentage of all malaria cases. Areas where incidence is >30% are considered 'high risk'. E, distribution of TNF enhancer haplotypes for the SNPs -1031T>C, -863C>A, -308G>A, -238G>A, -76T>C across Indian populations. The linguistic lineages (TB, Tibeto-Burman; IE, Indo-European; DR, Dravidian; AA, Austro-Asiatic) of populations are indicated. Endogamous populations that contributed to patient and control samples from the endemic and non-endemic region are marked by * and #, respectively in A and E. Blue and green circles in A represent patient recruitment sites in the *P. falciparum*-endemic and -non-endemic region, respectively.

Incidence of *P. falciparum *malaria in India is high with ~0.9 million cases reported annually in the last few years [9]. Several regions of the country are 'high risk' for *P. falciparum *with the infection accounting for more than 80% of all malaria cases in certain areas [10,11] (Figure 1d). The population of India represents largely endogamous groups comprising distinct religious communities, castes and isolated tribal populations. The Indian population is genetically heterogeneous as revealed by recent Indian Genome Variation Consortium (IGVC) data on 55 subpopulations of the country [unpublished data]. As differences in genetic structure (population stratification), selection and human adaptation are major confounders in association studies, it is important that case-control association studies for malaria in India be carried out on carefully defined, ethnically-matched sets of patients and controls.

Single nucleotide polymorphisms (SNPs) in the 5' regulatory region of *TNF *and coding region of *FCGR2A *have been associated with *P. falciparum *malaria [12-17]. Tumour Necrosis Factor (TNF) is involved in multiple inflammatory and immune responses and plays an important role in the pathogenesis of many infectious diseases including *P. falciparum *malaria [18]. The transcription of *TNF *is complex and tightly regulated [19,20]. SNPs in the 5' regulatory region of the gene have been shown to correlate with many infectious and inflammatory diseases [21,22] with conflicting reports regarding their functional significance. SNPs at positions -1031, -857, -376 (rs1800750, G>A), -308 and -238 in the proximal enhancer of the *TNF *gene exhibit differential associations to malaria and TNF production in different populations [12-14,23-25] suggesting that individual TNF responses may be genetically determined.

The human IgG receptor FcγRIIa (CD32) is an important link between the cellular and humoral arms of the immune system. It is a low affinity receptor for immunoglobulin subtypes IgG1–4 and also binds C-reactive protein (CRP) with high affinity. A polymorphism in exon 4 of *FCGR2A *(R131H, G/A) alters its function in vitro; the product of the G allele (131R) has preferential affinity for IgG1 and IgG3 while the A allele (131H) product binds efficiently to IgG2 while retaining its affinity for IgG1 and IgG3 [26]. The high-affinity IgG2 binding 131H allele has been correlated with susceptibility to severe *P. falciparum *malaria in Africa, particularly in children in Kenya and Gambia [15,16], but failed to show significant independent association with severity of malaria in Thai adults [27]. A recent report [17] has implicated the 131H allele in protection from severe malaria in Sudan.

Screening of the enhancer region of the *TNF *gene for genetic variants in the ethnically diverse Indian population and correlation of two SNPs (-1031 and -863) with circulating plasma TNF levels in healthy individuals and *P. falciparum *malaria patients is reported here. Correlation of TNF levels and 5' regulatory region SNPs with severity of disease was investigated in a case-control study with patients and controls drawn from a *P. falciparum *endemic and a non-endemic region of the country. The FcγRIIa R131H polymorphism was also evaluated for correlation with susceptibility to severe malaria in these regions.

## Methods

### Study subjects and sample collection

Analysis of allele frequency distribution of the selected *TNF *(RefSeq NT_007592.14) and *FCGR2A *(RefSeq NM_021642) SNPs was carried out in the existing Indian Genome Variation Consortium (IGVC) samples. This validation panel consisted of 1,871 samples drawn from 55 ethnically and linguistically diverse endogamous populations across India [28].

For the case-control study for an endemic (Antagarh, Chhattisgarh and Sundargarh, Orissa) and a non-endemic (Lucknow and surrounding areas of Uttar Pradesh) region (Figure 1a), approval was obtained from ethical committees of all participating institutions. Informed consent was obtained from each volunteer/guardian prior to collection. Blood samples (2 to 5 ml) were drawn from patients above five years of age diagnosed with *P. falciparum *malaria. Diagnosis was carried out by rapid diagnostic test kits (Optimal/Paracheck) followed by confirmation by examination of thick and thin film blood smears. Categorization of severe and non-severe cases was performed according to WHO guidelines [29]. Non-severe malaria patients were blood smear-positive, had fever and lacked symptoms that characterized severe malaria. Severe malaria cases were categorized as cerebral and non-cerebral. Cerebral malaria was characterized by impaired consciousness (coma) with fever. Any one of the following symptoms together with a positive blood smear indicated severe (non-cerebral malaria): severe anaemia, acidotic breathing, pulmonary oedema, hypoglycaemia, increased serum creatinine levels. A total of 121 patients (68 and 53 patients from the endemic and non-endemic region, respectively) and 100 healthy control individuals (42 and 58 uninfected individuals from the endemic and non-endemic region, respectively) were included in the study. Plasma TNF levels in the context of disease severity were analyzed for 175 patients (101 and 74 patients from the endemic and non-endemic region, respectively) and 186 control individuals (98 and 88 individuals from the endemic and non-endemic region, respectively). Control samples were taken from ethnically-matched, unrelated individuals from the endemic region and non-endemic region. Plasma was separated at the time of collection and stored in liquid nitrogen. G6PD level and HbA/S status of each individual was determined.

### Genomic DNA extraction and genotyping

Genomic DNA was extracted from peripheral blood leucocytes using salting-out procedure [30]. For validation of reported SNPs and discovery of novel SNPs, a 511 bp region of the TNF enhancer (-1 to -511) was sequenced in a discovery panel comprising 43 samples from different populations of India [28]. Genotyping of all *TNF *and *FCGR2A *SNPs in the IGVC validation panel was done by Sequenom mass spectroscopy. For patient and control samples, genotyping of -1031, -863 and -857 *TNF *SNPs was done by direct sequencing of a 292 bp PCR product covering nt -782 to -1073. Typing of other *TNF *SNPs (-308, -238, -76) and *FCGR2A *G/A (R131H) SNP was done by SNaPShot method (Applied Biosystems). All DNA sequencing and genotyping was performed on 3130xl automated DNA sequencer (Applied Biosystems). Approximately 10% genotypings carried out by SNaPshot were quality checked by DNA sequencing.

### ELISA for TNF

A sandwich ELISA was performed to measure plasma TNF levels using capture monoclonal anti-human TNF antibody (Pierce) and a paired biotinylated anti-human TNF antibody (Pierce). The minimum detection limit of the assay was 2 pg/ml.

### Statistical methods

The chi-square test was performed to evaluate whether the allele frequencies of the populations are in Hardy-Weinberg equilibrium. Comparison of TNF levels among genotypes of the -1031 and -863 SNPs was carried out by non-parametric Kruskal-Wallis test [31]. Pairwise comparison of means for TNF levels was carried out by Mann-Whitney test. Haplotypes for *TNF *SNPs were generated by PHASE. Linkage disequilibrium (LD) plot for *TNF *SNPs was generated using Haploview. Odds ratio (OR) for risk assessment was determined using EpiInfo™ version 3.4 software with the P-value calculated by Fisher Exact or Mantel-Haenszel test.

## Results

### *TNF *regulatory region and *FCGR2A *SNP frequencies vary across Indian populations

**Figure 2:**
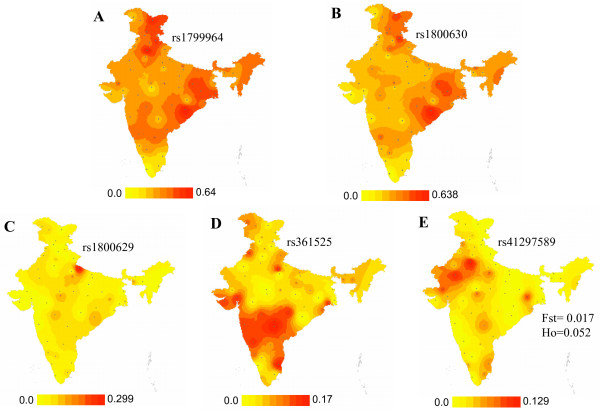
Minor allele frequency (MAF) distribution of *TNF *SNPs across India. A, rs1799964 (-1031); B, rs1800630 (-863); C, rs1800629 (-308); D, rs361525 (-238); E, rs41297589 (-76).

Validation of reported *TNF *5' regulatory region polymorphisms as well as discovery of possible novel SNPs in *TNF *promoter/enhancer was carried out by sequencing in representative samples of 43 populations of India (discovery panel) under the Indian Genome Variation Consortium (IGVC) [28]. A novel SNP, -76 T>A (allotted SNP ID: rs41297589), was discovered in the Indian population. Validation of the SNP in the larger panel comprising 1871 samples from 55 populations across India revealed an overall minor allele frequency (MAF) of 0.031. Forty populations, across all linguistic lineages, were polymorphic at this site (Figure 2). This SNP lies within the nt -131 to -63 region of the *TNF *enhancer that has been reported to be highly conserved in humans and primates [32] and is positioned at the low affinity transcription factor Ets-1 binding site in macrophages and the high affinity NFATp binding site in T and B cells [19]. Of the SNPs analyzed under IGVC, the *TNF *-76 T>A was among those exhibiting the lowest F_ST _and heterozygosity (H_o_) values (F_ST _= 0.017, H_o _= 0.052) suggesting negative selection. The distribution of *TNF *SNPs at positions -1031, -863, -308 and -238 across Indian populations is shown in Figure 1 and Figure 2. The frequency of the A allele of the *FCGR2A *exon 4 G/A R131H polymorphism across the 55 populations varied from 0.38 to 0.83. The distribution of the AA genotype is shown in Figure 1c. Allele frequency information for these SNPs across all populations may be accessed through the IGVC website [33].

This SNP frequency data was used to plan a case-control study to analyze the association of *TNF *and *FCGR2A *SNPs with *P. falciparum *malaria in India. Patient samples were collected from a *P. falciparum*-endemic area (Chattisgarh and Orissa) that has perennial and high malaria transmission [11] and a non-endemic region (Uttar Pradesh) with low and seasonal transmission. The main affected populations of the endemic region are isolated tribal groups of the Austro-Asiatic and Dravidian linguistic lineage while the patients in the non-endemic region are primarily from large populations (caste and religious groups) of the Indo-European lineage. Mean MAFs from populations of these regions covered by IGVC were compared (Table 1); allele frequencies of five populations from the non-endemic region (n = 197) and three populations from the endemic region (n = 86) were analyzed. Significant differences between the endemic and non-endemic region were observed for the *TNF *-1031 and -863 SNPs. The frequency of the *FCGR2A *(R131H) A allele was also higher in the endemic region although the P value was 0.07. Thus, the healthy control panel constituted for this study comprised ethnically-matched individuals from both regions. The combined control panel comprised equivalent numbers from both these groups and included populations represented in the patient pool.

**Table 1:** *TNF *enhancer and *FCGR2A *SNP frequencies in IGVdb populations from the *P. falciparum *endemic and non-endemic regions included in this study.

**Position**	**dbSNP ID**	**Minor allele frequency**		**P value**
***TNF***		**Non-endemic**	**Endemic**	
-1031 (T>C)	rs1799964	0.314	0.436	0.013
-863 (C>A)	rs1800630	0.242	0.405	0.003
-308 (G>A)	rs1800629	0.062	0.063	0.97
-238 (G>A)	rs361525	0.075	0.080	0.92
-76 (T>A)	rs41297589	0.030	0.054	0.338

***FCGR2A***				
Arg/His, G/A	rs1801274	0.568	0.66	0.07

### Homozygotes of *TNF *-1031C and -863A alleles are associated with elevated circulating TNF levels

The five *TNF *regulatory region SNPs described above as well as the -857 SNP previously correlated with cerebral malaria [13] were typed in patients and controls. As the -857, -308, -238 and -76 SNPs had relatively low frequency, only the -1031 and -863 SNPs were analyzed further.

Within the patient and control groups, significant differences in TNF levels were seen between genotypes of both -1031 and -863 (-1031, P = 0.0178 and 0.0089; -863, P = 0.0481 and 0.023 in controls and patients, respectively)(Table 2). Comparison between homozygotes indicated significant correlation of high TNF levels with the minor allele of both SNPs (P < 0.01) (Table 2). Although heterozygotes had higher levels than major allele homozygotes in both patients and controls, the difference was statistically non-significant.

**Table 2:** Correlation of TNF levels with genotypes of the -1031 and -863 SNPs in controls and patients. Comparison of means was carried out by Kruskal-Wallis test.

	**TNF levels as mean ± S.D. (pg/ml)**			
	
	**Controls (n = 100)**		**Patients (n = 111)**	
**-1031**				
TT	18.2 ± 22.17 (n = 58)	P = 0.0178	27.9 ± 29.14 (n = 58)	P = 0.0089
TC	21.3 ± 23.2 (n = 36)		40.9 ± 64.9 (n = 47)	
CC	56.56 ± 35.1 (n = 6)^a^		126.6 ± 80.82 (n = 6)^b^	
**-863**				
CC	19.01 ± 22.9 (n = 61)	P = 0.0481	25.6 ± 29.3 (n = 62)	P = 0.0231
CA	17.79 ± 17.9 (n = 30)		47.5 ± 66.8 (n = 44)	
AA	52.27 ± 35.1 (n = 9)^c^		124.7 ± 90.2 (n = 5)^d^	

'n' indicates number of individuals in each category.

^a,b ^TNF levels in TT vs. CC in controls and patients: P < 0.01 (Mann-Whitney test)

^c,d ^TNF levels in CC vs. AA in controls and patients: P < 0.02 (Mann-Whitney test)

**Figure 3:**
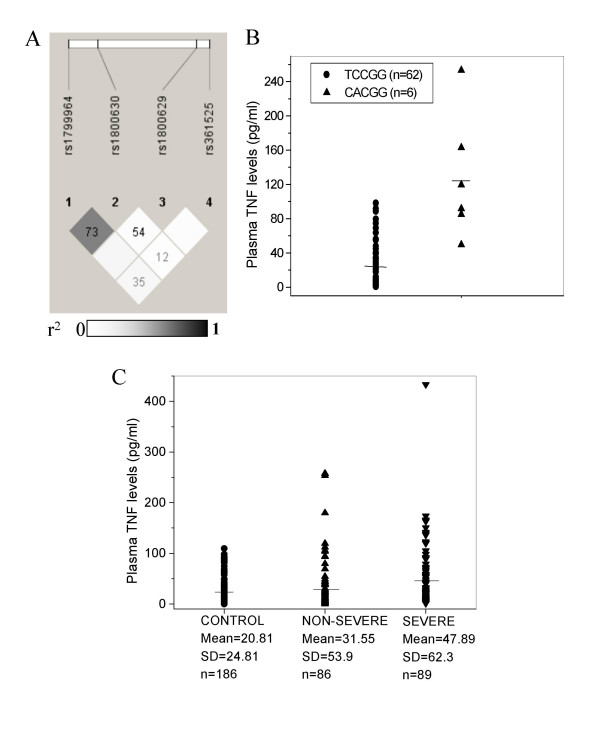
Linkage between *TNF *regulatory region SNPs and correlation of *TNF *haplotypes with plasma TNF levels. A, r^2 ^LD plot of four *TNF *SNPs (-1031, -863, -308, -238). Value in each cell is the percent D' between SNP pairs. B, Correlation of *TNF *promoter haplotype (-1031C, -863A, -857C, -308G, -238G) with elevated TNF levels compared to the major haplotype TCCGG. Mean plasma TNF levels were compared using Mann-Whitney test (z = -3.73, P = 0.0002). C, Plasma TNF level in patients and controls. Pairwise comparison of means by non-parametric Mann-Whitney test: severe versus control, z = 4.41, P < 0.0001; non-severe versus control, z = 0.67, P = 0.25; severe versus non-severe, z = 3.13, P = 0.0009.

The -1031 and -863 SNPs had a similar distribution pattern in Indian populations (Figure 1, Figure 2). Combined analysis of the patient and control groups indicated that alleles of -1031 and -863 were in linkage disequilibrium (LD) (D' = 0.73, r^2 ^= 0.494) while no linkage was observed with -308 and -238 (Figure 3a). Haplotype analysis of the control groups revealed that the CACGG haplotype (-1031C, -863A, -857C, -308G, -238G) in which both -1031 and -863 were mutated had the highest frequency (0.115) after the major haplotype TCCGG (0.58). The CAGGT haplotype (-1031C, -863A, -308G, -238G, -76T), polymorphic at -1031 and -863, was the predominant haplotype after the major haplotype TCGGT in the IGVdb populations as well (Figure1e). Thus, the possible correlation between TNF levels and the CACGG haplotype was investigated by comparing TNF levels in subjects that were homozygous for the CACGG haplotype and the major haplotype TCCGG. Individuals homozygous for CACGG from both patient and control groups had significantly higher TNF levels than those homozygous for TCCGG (P = 0.0001) (Figure 3b) indicating strong association of the haplotype with elevated TNF levels.

### High TNF levels correlate with severe *P. falciparum *malaria

Elevated TNF levels in malaria patients have been correlated with severe disease manifestation in other world populations [34,35] although significant differences between plasma TNF levels were not observed between uncomplicated, severe anemia, and cerebral malaria patients in a recent report on Malian children [23] suggesting the possibility of population-specific differences. An earlier study from India [36] reported higher TNF level in patients with multiple organ dysfunction and those who died. Our results indicated significantly higher TNF levels in patients with severe *P. falciparum *malaria when compared with controls (P < 0.0001) or with patients with non-severe malaria (P = 0.0009) (Figure 3c). The difference in TNF levels between non-severe patients and controls was not significant (P = 0.25). Significant differences were not seen between non-cerebral severe and cerebral malaria patients (P = 0.102) suggesting correlation with severe disease manifestation including cerebral malaria.

### Correlation of *TNF *and *FCGR2A *SNPs with susceptibility to severe *P. falciparum *malaria

The -1031C allele was associated with disease severity with higher percentage of the mutant genotypes observed in severe patients compared with controls (55.5% versus 42%, OR = 1.76, 95% CI = 0.97–3.2, P = 0.048) (Table 3). A similar trend was observed for the -863A allele (51.7% versus 39%, OR = 1.69, 95% CI = 0.93–3.09, P = 0.065). Significant difference in distribution of the minor allele for both -1031 and -863 was also observed between severe and non-severe patient groups (-1031, 55.6% versus 40.4%, OR = 1.87, 95% CI = 1.03–3.4, P = 0.028; -863, 51.85% versus 36.8%, OR = 1.84, 95% CI = 1.01–3.38, P = 0.033) indicating association of the minor allele with disease severity. Interestingly, when severe cases were analyzed separately as non-cerebral severe (NCM, n = 33) and cerebral malaria (CM, n = 21), stronger association of the -1031C allele was indicated with non-cerebral severe malaria as compared to cerebral malaria (CM versus non-severe, OR = 1.38, 95% CI = 0.76–2.52, P = 0.25; NCM versus non-severe, OR = 2.25, 95% CI = 1.23–4.13, P = 0.004) suggesting association of the minor allele with non-cerebral but not cerebral severe disease manifestation. Taken together, our data indicated a strong correlation between the *TNF *-1031 and -863 SNPs and cytokine levels in individuals, association of high TNF level with severe malaria as well as correlation of -1031C and -863A polymorphisms with disease severity.

**Table 3:** Genotype distribution of *TNF *regulatory region SNPs in controls and patients.

	**Control (n = 100)**	**Non-severe (n = 57)**	**Severe (n = 54)**
**-1031**			
TT	58 (58%)	34 (59.6%)	24 (44.4%)
TC	36 (36%)	21 (36.8%)	26 (48.1%)
CC	6 (6%)	2 (3.5%)	4 (7.4%)
**-863**			
CC	61 (61%)	36 (63.1%)	26 (48.1%)
CA	30 (30%)	19 (33.3%)	25 (46.2%)
AA	9 (9%)	2 (3.5%)	3 (5.5%)

**Figure 4:**
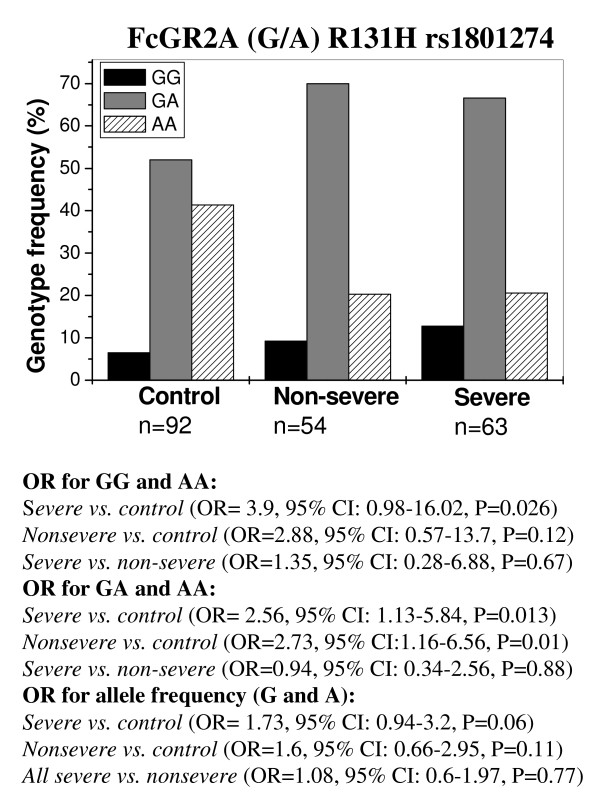
Correlation of *FCGR2A *G/A R131H polymorphism with *P. falciparum *malaria in combined analysis of samples from the endemic and non-endemic region. Genotype frequency distribution in controls and patients is depicted. Odds ratios (OR) indicate association of the G allele with increased risk of malaria.

In light of the differential association reported for the *FCGR2A *G/A (R131H) polymorphism with *P. falciparum *malaria in different populations, we analyzed its distribution in patient and control groups. Combined analysis of endemic and non-endemic region samples indicated that the high affinity IgG2-binding 131H allele associates with protection from disease manifestation; the GG homozygote for 131R correlated with susceptibility to severe malaria (severe vs. control, OR for GG and AA = 3.9, P = 0.026) (Figure 4). Heterozygote for the G allele also correlated with manifestation of disease (severe vs. control, OR for GA and AA = 2.56, P = 0.013; non-severe vs. control, OR for GA and AA = 2.73, P = 0.01) (Figure 4). No significant difference was observed in the distribution of GA and GG genotypes in different disease groups (P > 0.05 in all odds ratio calculations). The 131H/H genotype was associated with protection with the control group having a significantly higher proportion of the AA genotype compared to the non-severe (41.3% versus 20.3%, OR = 2.78, 95% CI = 1.41–5.5, P = 0.001) and severe (41.3% versus 20.6%, OR = 2.61, 95% CI = 1.34–5.13, P = 0.002) patient groups. After stratifying for populations in the endemic and non-endemic region, a stronger association of the G allele with severe malaria was observed in the non-endemic region (severe vs. control, OR for GG and AA = 4.67, 95% CI = 0.75–33.94, P = 0.047 in non-endemic region and OR for GG and AA = 2.83, 95% CI = 0.17–33.76, P = 0.32 in endemic region; severe vs. control, OR for GA and AA = 2.59, 95% CI = 0.93–7.4, P = 0.043 in non-endemic region and OR for GA and AA = 2.43, 95% CI = 0.5–12.06, P = 0.178 in endemic region). Our results indicate that the 131R allele is associated with susceptibility to disease with the 131H/H genotype being protective against *P. falciparum *malaria and suggest differences in FcγRIIa R131H allelic association profiles in regions with differential *P. falciparum *prevalence and transmission.

## Discussion

The role of TNF during *P. falciparum *malaria infection has been described as both protective and pathogenic [37,38]. At low levels, TNF is believed to augment parasite killing by macrophage activation and subsequent release of cytokines, whereas high TNF level has been associated with severe manifestations like acute respiratory distress and cerebral malaria. A recent study on lethal malaria in mice has implicated high levels of TNF in impairment of dendritic cell function thus contributing to immunosuppression associated with malaria [39]. Individual variation in TNF production mainly by macrophages and NK cells is likely to influence severe disease manifestation. Although the effect of *TNF *enhancer SNPs on transcription levels of the cytokine remains controversial [40,41], several studies have implicated their role in determining TNF levels in individuals and consequently influencing their response to a gamut of autoimmune and infectious diseases including *P. falciparum *malaria [42,43]. While association of the -863A substitution with reduced TNF levels was described in the Swedish population [44], a study from Japan [45] reported association of the -1031, -863 and -857 polymorphisms with increased reporter gene expression and increased concanavalin A-stimulated TNF production from peripheral blood mononuclear cells. Additionally, the ubiquitous transcription factor OCT-1 has been reported to exhibit allele-specific binding to the variant allele -863A or -857T [46]. In our study as well, higher circulating TNF levels in both controls and patients were associated with the -1031C and -863A polymorphisms. The two SNPs were in LD and the CACGG haplotype (-1031C, -863A, -857C, -308G, -238G) was strongly associated with higher plasma TNF levels. This haplotype had the highest estimated frequency in controls (0.115) after the major haplotype TCCGG (0.58). As the -1031 and -863 polymorphisms are present in high frequency in India and the CAGGT haplotype (-1031C, -863A, -308G, -238G, -76T) is also the predominant haplotype after the major haplotype TCGGT in most of the 55 IGVdb populations analyzed by us, the two SNPs may play a significant role in modulating the immune response and influencing the outcome of several infectious diseases.

Our results indicated association of the -1031 and -863 *TNF *SNPs with increased risk of severe malaria. A recent study on Thailand/Myanmar border populations reported association of the *TNF *-1031C, -863C, -857C allele with severe malaria [14]. This allele was not in significant LD with HLA-DRB1 and HLA-B alleles indicating that association with susceptibility to cerebral malaria was not because of LD with the HLA alleles. To our knowledge, our study is the first report of independent association of the -863 polymorphism with increased risk of severe disease.

In contrast to previous reports [15,16,27], our data implicates the low-IgG2 binding 131R allele in susceptibility to *P. falciparum *malaria with the high-affinity IgG2 binding 131H/H genotype being associated with protection from disease. Our results are in agreement with a recent study on subjects from Sudan that demonstrated that the 131R/R genotype and the 131R allele were significantly associated with the odds of severe malarial disease [17]. The 131H/H genotype was associated with a reduced risk of both non-severe and severe malaria in our study, indicating a role for the polymorphism in protection from clinical manifestation of disease. The FcγRIIa 131H receptor binds IgG3 more efficiently than 131R and is the only high-affinity receptor for IgG2 [47,48]. Cytophilic antibodies are thought to be critical in protective immunity and several studies have shown an association between increased levels of IgG1 and IgG3 subclasses and protection from malarial infection or disease [49-51]. High levels of IgG2 antibodies to MSP-2 and RESA have been associated with resistance to *P. falciparum *malaria in Burkina Faso [52]. IgG2 antibodies may be cytophilic in individuals carrying the 131H allele as these bind Fcγ receptor with greater affinity. Nasr et al. [17] showed that IgG3 antibodies specific to crude malarial antigen were more associated with reduced risk of clinical malaria in 131H/H individuals than in 131R/R individuals; weak but statistically significant association between low levels of anti-malarial IgG2 antibodies specific to a recombinant antigen in 131H allele carriers and reduced risk of severe malaria was also found. Our results also suggest a role for the 131H/H genotype in protection against clinical malaria. This may be mediated by enhanced phagocytosis of *P. falciparum*-infected erythrocytes by cells expressing the 131H receptor [53] or by FcγRIIa and FcγRIII-dependent parasite neutralizing activity of monocytes during antibody-dependent cell-mediated inhibition of parasite growth [54].

A difference in the degree of association of the FcγRIIa 131R allele with severe malaria observed in the two regions included in our study may be a reflection of the nature of immune response against malaria in areas characterized by low and seasonal transmission or endemic areas with high transmission rates. Although a more detailed analysis of this is warranted, it may be relevant that association of high IgG2 levels with protection from severe *P. falciparum *malaria observed in the Burkina Faso study [52] as well as the association of the low IgG2 binding 131R allele with disease severity in a Sudanese population [17] were both carried out from areas characterized by seasonal *P. falciparum *malaria transmission. As evident from the map of *P. falciparum *incidence in India (Figure 1d) and the distribution of the FcγRIIa AA genotype (Figure 1c), higher frequency of the protective 131H/H genotype was observed in 'high risk' and endemic areas of east and north-east India. This is suggestive of possible selection of the protective A allele in populations exposed to *P. falciparum *malaria in these regions.

## Conclusion

In light of the increasing incidence of *P. falciparum *malaria and its concentration in specific regions of India, we initiated a case-control study to understand the role of genetic variants of specific immune regulatory molecules in malaria severity/resistance in an endemic and non-endemic region of the country. Differences in frequencies of *TNF *enhancer and *FCGR2A *SNPs were observed in populations from the two regions. Association of specific *TNF *and *FCGR2A *SNPs with cytokine levels and disease severity/resistance was indicated in patients. The *TNF *enhancer haplotype carrying the -1031 and -863 SNPs is the predominant haplotype after the major haplotype in most Indian populations and correlated with enhanced TNF levels. In addition, the FcγRIIa 131R low affinity IgG2-binding allele was associated with susceptibility to disease with the 131H/H genotype being protective against *P. falciparum *malaria. Differences in FcγRIIa allelic association profiles were observed in regions with differential *P. falciparum *prevalence and transmission. The data underlines the need for addressing the contribution of human genetic factors in malaria in the context of disease epidemiology and population genetic substructure within India.

## Abbreviations

TNF, tumour necrosis factor; FcγRIIa, Fc gamma receptor IIa; SNP, single nucleotide polymorphism; MAF, minor allele frequency; ELISA, enzyme-linked immunosorbent assay.

## Authors' contributions

SH and VV conceived and designed the study and contributed to execution of research. SH and S Sinha wrote the manuscript. S Sinha, SKM, and S Sharma carried out genotyping, ELISAs, and data analysis and contributed to sample collection and processing. PKP, PKM, SKS, RMB, HJ and APD helped in design and implementation of field studies. SM, SSP, SKM, BKR, RCA and SA are clinicians who contributed to diagnosis and categorization of falciparum malaria patients. The Indian Genome Variation Consortium (IGVC) comprises scientists from six institutes of the Council for Scientific and Industrial Research with Dr. S.K. Brahmachari, Institute of Genomics and Integrative Biology, New Delhi as coordinator. Collection of endogamous population samples across India and Sequenom genotyping data analyzed here was coordinated under IGVC. SH is a member of the consortium. All authors have read and approved the final manuscript.
